# The Perfect Burrow, but for What? Identifying Local Habitat Conditions Promoting the Presence of the Host and Vector Species in the Kazakh Plague System

**DOI:** 10.1371/journal.pone.0136962

**Published:** 2015-09-01

**Authors:** Bethany Levick, Anne Laudisoit, Liesbeth Wilschut, Elisabeth Addink, Vladimir Ageyev, Aidyn Yeszhanov, Valerij Sapozhnikov, Alexander Belayev, Tania Davydova, Sally Eagle, Mike Begon

**Affiliations:** 1 Ecology, Evolution and Genomics of Infectious Disease Research Group, Institute of Integrative Biology, University of Liverpool, Liverpool, United Kingdom; 2 Department of Physical Geography, Utrecht University, Utrecht, The Netherlands; 3 M.Akimbayev’s Kazakh Science Centre for Quarantine and Zoonotic Diseases, Almaty, Kazakhstan; 4 Taldykorgan anti-plague station, Taldykorgan, Kazakhstan; 5 Institute of Infection and Global Health, University of Liverpool, Liverpool, United Kingdom; USGS National Wildlife Health Center, UNITED STATES

## Abstract

**Introduction:**

The wildlife plague system in the Pre-Balkhash desert of Kazakhstan has been a subject of study for many years. Much progress has been made in generating a method of predicting outbreaks of the disease (infection by the gram negative bacterium *Yersinia pestis*) but existing methods are not yet accurate enough to inform public health planning. The present study aimed to identify characteristics of individual mammalian host (*Rhombomys opimus*) burrows related to and potentially predictive of the presence of *R.opimus* and the dominant flea vectors (*Xenopsylla spp.*).

**Methods:**

Over four seasons, burrow characteristics, their current occupancy status, and flea and tick burden of the occupants were recorded in the field. A second data set was generated of long term occupancy trends by recording the occupancy status of specific burrows over multiple occasions. Generalised linear mixed models were constructed to identify potential burrow properties predictive of either occupancy or flea burden.

**Results:**

At the burrow level, it was identified that a burrow being occupied by *Rhombomys*, and remaining occupied, were both related to the characteristics of the sediment in which the burrow was constructed. The flea burden of *Rhombomys* in a burrow was found to be related to the tick burden. Further larger scale properties were also identified as being related to both *Rhombomys* and flea presence, including latitudinal position and the season.

**Conclusions:**

Therefore, in advancing our current predictions of plague in Kazakhstan, we must consider the landscape at this local level to increase our accuracy in predicting the dynamics of gerbil and flea populations. Furthermore this demonstrates that in other zoonotic systems, it may be useful to consider the distribution and location of suitable habitat for both host and vector species at this fine scale to accurately predict future epizootics.

## Introduction

Despite the relative rarity of plague (*Yersinia pestis* infection) in modern human populations, historical outbreaks such as the Black Death across medieval Europe clearly demonstrate the disease’s devastating potential [1]. Now largely confined to foci in wildlife populations, plague has been characterised as an emerging (or re-emerging) infectious disease [2]. Emerging infectious diseases and in particular zoonotic infections from wildlife species are considered a great future danger to human health worldwide [3], with their emergence being linked to both multiple socio-economic factors and environmental conditions [4]. Being able to increase our ability to predict and prevent epizootics, and in particular minimising the risk of spillover into human populations, is essential for controlling these emerging diseases.

The plague system in the Pre-Balkhash region of Kazakhstan has been subject to extensive study over the past 15 years. The desert and semi-desert belt across the region consists of a vast area of mixed vegetation and shrubs that extends from the Caspian Sea in the west to the Chinese border in the south east of the country, in the Almaty province. The northern desert subzone of the Almaty province is the largest of 20 endemic plague foci in the country. The hosts from this focus have been recently suggested to have had involvement in initiating the European outbreaks of plague in the 1300’s [5]. However, since the 1950’s the national (previously USSR) anti-plague system has been largely successful in preventing the occurrence of human cases [6, 7]. Across 14 of the foci located in the Pre-Balkash region, plague is mainly transmitted between great gerbils (*Rhombomys opimus*) by a variety of fleas, although *Xenopsylla spp.* generally dominate. *R.opimus* is the dominant rodent in the landscapes of the Pre-Balkash area, inhabiting complex burrow systems as extended family groups. Previous work has identified that plague spreads through gerbil populations in a manner well described by percolation theory [8]. Importantly, this model followed previous work [9] in predicting a critical threshold of gerbil abundance (defined as the proportion of gerbil burrow systems that are occupied) necessary for a plague epizootic to occur. A more recent adaptation of the model extends this to describe the threshold as a curve arising as a function of both the abundance of the gerbils and their flea burden [10]. However the dynamics of plague transmission, and of the gerbil population, have not been considered previously at the scale of the individual burrow.

*R.opimus* burrows are visible on satellite images due to depletion of vegetation in the active (or so-called ecological) centre of the burrow, and the surrounding area where activities are most intense [8, 11, 12]. Like many other diurnal rodents, their complex and extensive burrow systems are important for their social behaviours [13] and provide nesting and food chambers, safe access to above ground food resources and refuges from predators [14]. The great gerbil’s burrowing activities promote the “fertile island effect” in influencing the concentration of soil nutrients and available nitrogen [15] and as such the great gerbil can be considered as a powerful ecosystem engineer, capable of modifying physio-chemical properties of the soil at the fine burrow system scale. These burrows also provide a buffered environment and refuge for a number of invertebrate species, in particular those that feed on gerbils and are potential vectors (mainly fleas and ticks) of pathogenic agents. The number, position, and size of the burrow systems generally do not change over time, but the proportion of burrow systems occupied by family groups may fluctuate dramatically hence making them an effective proxy for great gerbil abundance [8, 9].

The ultimate aim of the present study is to improve our ability to predict plague incidence in the Pre-Balkhash region, through increased ability to predict the presence of the rodent host and the abundance of the flea vector at the level of the individual burrow. Given their importance for the presence of plague, we can consider that a burrow that is highly preferred by both the host and vector may be a perfect burrow for *Y.pestis*. Further, for plague to spread across a landscape, burrows occupied by the host must be distributed with sufficient connectivity to permit percolation through the population. Preference for burrows with specific attributes may cause clustering of burrows supporting gerbils and fleas within the landscape, with extensive gaps in between, potentially preventing long distance spread of plague [16].

Descriptive accounts of *R.opimus* burrow properties and ecological preferences have been reported previously [17]. More recent work has identified landscape-scale properties with functional corridors and barriers for potential plague transmission; that is, areas of high and low density of burrows (though all burrows were considered rather than those occupied by gerbils or fleas; [18]). However, as yet there has been no quantitative analysis of landscape features at the level of the individual burrow that are associated with their being occupied, with remaining occupied over a number of seasons, or with gerbils in the burrow having a high flea burden. Such is the aim of the present study.

## Methods

### Study Site

The study sites are located in the Pre-Balkhash endemic plague focus in south eastern Kazakhstan (ranging between 44°75′43.61 and 45°73′73.80; see also [12]). The cartographic division of the territory for plague monitoring into primary squares (40km x 40km), and then each into four secondary squares (20km x 20km) and these into four sectors (10km x 10km), as well as the large scale features of the landscape, have been described elsewhere [8, 11, 18, 19]. The study sites were located in 6 sectors on the east bank of the Ili River within the alluvial plains that follow the historical delta in the northern portion of the focus [20]. The semi-desert vegetation consists of short grass (Gramineae, Poaceae), herbs (mainly Chenopodiaceae, halophytic vegetation) and shrubs dominated by *Haloxylon* and *Calligonum* species.

In four seasons (monitoring periods of around 1–2 months; autumn (September) 2011, summer (July) 2012, autumn (September) 2012 and spring (April-May) 2013), all burrow systems located within randomly chosen 500m x 500m (25ha) squares spread over the 6 sectors were mapped. In total, 2113 burrows were mapped. Of these, occupancy status was recorded twice (in two seasons) for 894 burrows and just once for 1219 burrows. The GPS location of the burrow system was recorded along with its altitude and its occupancy status.

Individual burrow systems were identified by locating the central burrow structures and the outer limits of each discrete burrow unit (typically around 50m in diameter). For each burrow a description of its occupancy status and the landscape of the area surrounding the burrow was created. If direct evidence of gerbils was found at the burrow (fresh faeces, obvious digging) then it was classified as occupied. Signs of gerbil activity limited to feet/tail markings in the sand at the entrances of burrows would classify a burrow as visited. If no gerbil activity was identified then the burrow was classified by its condition; if in usable condition but otherwise not inhabited the burrow was classified as unoccupied, varying states of disrepair would identify the burrow as old or disappeared.

A landscape description of the burrow was then generated. This was based on visual inspection of landscape properties of the local area in which the burrow had been constructed, in particular guided by granulometry to classify sediments. Classification of landscape properties was thus conducted by methods detailed in Pogrebinsky (1963) [20, 21] and those specifically relating to the granulometry techniques are detailed further in Wentworth 1992 [22]. The properties, recorded for each burrow, were split into sediment properties of which there were eight (presence of sand, clay etc.), and topographical features of which there were seven (position on a dune, waterlogged areas, etc.). These characteristics and their recording is detailed in the supplementary material (Table I in S1 File).

During daylight hours, great gerbils (*R.opimus*) were caught using local traps placed at burrow entrances; trapping procedures and laboratory protocols were performed according to Russian standard methods [23, 24]. Gerbil traps were visited frequently so as to minimise their time in a trap (typically less than thirty minutes). Following this they were euthanased according to Kazakh public health protocols, as approved by the University of Liverpool Animal Welfare Committee. Trapped rodents were brought to the laboratory for processing. Individual rodents were sexed, weighed, and classified as juvenile or adult. For each rodent, fleas and ticks brushed off rodent fur were identified to species (fleas) or genus (tick) level, counted, sexed, and crushed in saline (NaCl 1%), before plating on selective agar to attempt to isolate *Y.pestis*. Further details of the laboratory techniques can be found in the supplementary material (S1 File). The number of such fleas collected from a rodent trapped in a burrow divided by the number of rodents trapped from the burrow is referred to as the flea index (and tick index was computed similarly).

A second data set (hereafter referred to as the long term data set) was collected to examine occupancy over multiple seasons. Methods differed little from those above except that the squares monitored were 200m x 200m, and rodents were not trapped. 513 unique burrows were revisited in each of six seasons (spring, summer and autumn 2011; spring and summer 2012; spring 2013) and their occupancy recorded each time. Some of these burrows were trapped as per the protocol above in Spring 2013 and these records added to the main data set.

### Ethics

All animal trapping and handling procedures were standard practice for Kazakh fieldworkers, requiring no specific permission and none involved endangered or protected species. These procedures were additionally approved by the University of Liverpool Animal Welfare Committee.

### Statistical methods

#### Generating landscape factors

A unique burrow landscape description was generated for each of the 2113 mapped burrows. Some of the landscape characteristics are mutually exclusive, but many can occur together and so as many characteristics as were needed to fully describe the burrow were noted in each case. The resulting data set was comprehensive and highly detailed, but the large variety (171 unique descriptions) of individual burrow landscape descriptions required condensing in order to identify key aspects of the landscape linked with the outcome variables (occupancy, long term maintenance of occupancy, burden of fleas).

Landscape descriptions could be considered at their finest scale (e.g. the burrow is on the side of a dune, on the top, or on the very bottom) or at a coarser level (the burrow is on a dune). There was little *a priori* indication of the coarseness of landscape description relevant to the outcome variable. Therefore an initial exploratory quantitative analysis, designed specifically for this data set, was conducted to identify general trends in the outcome variables across different landscape descriptions and groups of characteristics. For the two general outcome variables (occupancy levels, fleas) the level of an outcome response associated with each of the burrow landscape descriptions across the data set was estimated. For fleas this was measured as the mean flea index associated with burrows possessing each landscape description, and for occupancy as the proportion of burrows possessing this description that were occupied. In each case this was normalised by the number of burrows possessing each description to avoid over-representation of the effect of burrow properties that were simply common throughout the landscape.

All of the unique individual burrow descriptions in the data set were ranked by their level of outcome response (normalised by the number of burrows possessing this description). The ranked descriptions were then split into a high, medium and low group by their associated response level. These groups each contained roughly one third of the descriptions, with some allowance for groups of descriptions with the same level of response to be grouped together. For each of these groups, the proportion of descriptions in the group containing each of the 15 characteristics was calculated. Where the proportion of descriptions containing a particular characteristic differed by approximately 5–20% between the high and medium or low and medium groups, the characteristic was considered either as a predictive landscape factors to be used in statistical analysis or for inclusion within a predictive factor, (for more details see the supplementary document (S1 File)). In the latter case, where landscape characteristics describing related properties showed similar relationships with the outcome variable, they were grouped together to form landscape factors to be used in the statistical analysis. One such case, for example, were the landscape descriptors relating to the burrow being located on a dune (D, E, F, 7 & 8), including descriptions of the position on the dune and the stability of the dune structure, but which were ultimately grouped together into the “dune” factor. The resulting landscape factors, overall, are detailed in Table 1.

**Table 1:** Landscape factors used in models, built using landscape descriptors listed above.

Model variable	Nature of variable
Sand	Binary, presence or absence of sandy soils
Clay	Binary, presence or absence of clay soils
Loam	Binary, presence or absence of loamy soils
Solonchak/takir	Binary, presence or absence of solonchak/takir areas
Dunes	Binary, presence or absence of dunes

#### Statistical Models

Generalised linear mixed models (GLMMs) were used to explore the relationship of the model variables described in Table 2 with both occupancy status and flea burden. In general, the models were used to test hypotheses regarding putative predictors identified from field observations and the biology of the system, detailed below in Table 2.

**Table 2:** Landscape factors and further environmental factors tested in each model.

Response variable	Landscape factors tested	Environmental factors tested
Occupancy	Solonchak, sand, loam, clay, dunes/clay * dunes	Latitudinal position, season, tree presence
Long term occupancy	Solonchak, sand, loam, clay, dunes/clay * dunes	Latitudinal position, season, tree presence
Flea burden	Sand, loam, clay, dunes	Latitudinal position, season, tree presence, tick index, burrow occupancy in surrounding sector that season
*Xenopsylla* burden	Sand, loam, clay, dunes	Latitudinal position, season, tree presence, tick index, burrow occupancy in surrounding sector that season

In particular for models of occupancy, apart from individual landscape factors, the interaction (and respective main effect terms) between burrows being on clay sediment and the presence of dunes was included to test the hypothesis that their potential advantages (for clay sediment: stability; for dunes: ease of digging, an angled entrance providing extra shelter from wind/rain) would make their combined presence of greater benefit. Tree presence was included to test the hypothesis that trees are indicators of moisture concentration in the environment, and therefore in turn indicate fertile areas with ready food supply. For models of flea burden, the tick index was tested as a predictor to test whether certain burrows were more likely to be generally infested with invertebrates. In addition the percentage of burrows occupied in the surrounding sector (in the same season as the flea record was taken) was tested as a predictor of flea index to identify whether the flea burden was related to gerbil abundance.

#### Predicting Occupancy

The field recording of occupancy status was condensed to a binary record of occupied or unoccupied (the latter including all other recorded statuses; visited, empty or old). GLMMs using a binomial error distribution were constructed to predict occupancy for the full data set of 3011 observations (i.e. trapping events) of 2113 unique burrows, including repeat visits. Additionally, as multiple visits to sectors were not randomised, both burrow identity and sector were included as random effects (with burrow identity nested by their relevant sector).

A second GLMM was constructed to identify factors predictive of burrows remaining occupied using the long term occupancy data set, using, as the dependent variable, the proportion of the six monitoring periods where each of the revisited burrows was occupied. As above, sector was included as a random effect. The same selection of landscape factors was used in both cases.

#### Predicting flea numbers

The data set contained 999 observations of flea burden, of which *Xenopsylla spp.* dominated as anticipated (making up 98.9% of the observations). A GLMM was constructed using records of flea burden, using a poisson error distribution, and using a quasi-poisson model as a correction of standard errors due to over dispersion in the data [25], while including burrow and sector as nested random effects (as in the static occupancy model above).

#### Model reduction and comparison

In each case, a model was first constructed using all of the burrow level landscape traits hypothesised to be related to the outcome. This model was minimised as detailed below. A second model was then built using the significant landscape factors with the addition of further environmental factors, as detailed in Table 3. Again, this was minimised by the same process. Akaike’s Information Criterion (AIC) scores were estimated for the occupancy and long term occupancy models by finding the AIC score for the non-mixed version of the model (i.e. without random effects). For the models with quasipoisson error corrections, a quasi-AIC (qAIC) score was estimated, using a non mixed version of the model to obtain a dispersion parameter, and a model without error corrections to obtain a likelihood score [26]. Models with a ΔAIC of less than two were considered to have equal explanatory ability [27]. Where all factors were significant, backwards elimination was applied to identify any significant factors whose removal may improve the model AIC score. This was not performed in the flea models due to the additional complexity (and associated uncertainty) in the methods used to obtain the AIC estimates as detailed above.

**Table 3:** Description of terms in the parsimonious model of occupancy status.

Factor	Description of relationship	Coefficient	P Value	Standard Error
Latitudinal position	Occupancy higher with increasing latitude (to the north)	2.14	<0.01	0.41
Tree presence	Occupancy higher in the presence of trees	1.19	<0.01	0.12
Season (Summer 2012)	Occupancy lower than the reference of Autumn 2011	-0.35	<0.03	0.16
Season (Autumn 2012)	Occupancy lower than the reference of Autumn 2011	-0.45	<0.05	0.18
Season (Spring 2013)	Occupancy lower than the reference of Autumn 2011	-0.95	<0.01	0.11
Clay	Occupancy lower in presence of clay	-0.42	<0.01	0.13

However, while the AIC scores provide a method of comparing the different models, in each case they are estimated for the non-mixed version of the model and so we consider them to be approximate measures of the fits of the models with random effects included. They were therefore considered alongside the complexity of the model and the significance of the parameters. Specifically, simpler models, and those where all terms were significant, notwithstanding AIC score, were prioritised as detailed below.

In the final parsimonious static occupancy and flea models, checks of the nested random effects structure were performed by iteratively running the model, using a subset of the data consisting of one record from every instance where a burrow was sampled more than once, alongside all records of burrows only recorded on one occasion. The range and average of the coefficients and P values in these models were compared to those produced by the models using the nested random effects (see also [28]). These results are shown in the supplementary material. Only where effects from the original models were confirmed by this alternative were results considered reliable (details below).

All statistical analysis was conducted using the R statistical computing environment [29], using the additional package MASS [30].

## Results

### Occupancy status

Of the terms included in the landscape factor model, the interaction term between clay sediments and dunes was found to be significant. However the ΔAIC between the models with and without the interaction term did not differ significantly (AIC with interaction = 4032.86, without interaction = 4032.74). Hence, the simpler model without the interaction term was chosen as a minimal landscape model. Within this only the clay sediment term was significant and this was carried forward to the second model. Of the additional terms then tested, latitudinal position, the presence of trees and season were found to be significant. Backwards selection did not suggest removal of any of these would create significant improvement in the AIC scores, and as all terms were significant this was maintained as the minimal model. All of the model terms were validated by the iterative sampling method (S1 File). Descriptions of these relationships, and their associated coefficients are detailed in Table 3.

### Long term occupancy

The same landscape factors were tested as in the occupancy model. The interaction term between clay sediment and dunes, and its constituent main effects were found to be significant alongside sand. Here the model with the interaction had a significantly lower AIC (without interaction = 2155.80, with interaction = 2090.71) compared to the simpler model with just the main effects, and so as the terms were significant this was taken as the minimal landscape model. Of the additional environmental factors tested alongside these landscape factors, only latitude was found to be significant. The model including the non-significant environmental terms (season, presence of trees) was significantly better in terms of AIC (1625.00 with insignificant terms, 1770.00 without), but the reduced model was selected due to its increased simplicity and exclusion of insignificant terms. Again, backwards selection did not indicate that removal of any of these terms would significantly improve the model. Descriptions of these relationships, and their associated coefficients are detailed in Table 4.

**Table 4:** Description of terms in the parsimonious model of multiple season occupancy status.

Factor	Description of relationship	Coefficient	P Value	Standard Error
Latitudinal position	Burrows in northern regions occupied for a higher proportion of seasons	3.55	<0.01	0.44
Sand	Burrows occupied for a higher proportion of seasons in the presence of sand	0.67	<0.01	0.68
Dunes	Burrows occupied for a higher proportion of recorded seasons in the presence of dunes	0.23	0.25	0.20
Clay	Burrows occupied for a higher proportion of recorded seasons in the presence of Clay	0.41	0.12	0.26
Dunes * Clay	Burrows occupied for a lower proportion of recorded seasons when both are together	-1.73	<0.01	0.49

### Flea burden

Of the four landscape factors tested, only clay was found to be a significant predictor. Of the additional environmental factors alongside clay, only the tick burden and season were found to be significant. The model including all of the additional environmental factors (latitudinal position, tree presence, surrounding burrow occupancy) had a significantly lower AIC (with all factors = 568, with only significant factors = 571), but the simpler model with only significant parameters was taken as the final parsimonious model as detailed in Table 5. Again, all of the model terms were validated by the iterative sampling method (S1 File).

**Table 5:** Description of terms in parsimonious model of *Xenopsylla* burden.

Factor	Description of relationship	Coefficient	P Value	Standard Deviation
Tick burden	*Xenopsylla* burden higher where tick burden is higher	0.02	<0.01	0.00
Season (Summer 2012)	*Xenopsylla* burden higher than in reference season of Autumn 2011.	0.16	0.12	0.01
Season (Autumn 2012)	*Xenopsylla* burden lower than in reference season of Autumn 2011	-0.31	<0.01	0.01
Season (Spring 2013)	*Xenopsylla* burden lower than in reference season of Autumn 2011	-0.02	0.78	0.09

## Discussion

### Occupancy

Here we have identified that both static gerbil occupancy, and the maintenance of burrow occupancy in the long term are related to the burrow’s latitudinal position. Further, static occupancy is found to be related to the presence of trees, clay soils and the season, and the maintenance of occupancy is related to the presence of sand, and an interaction between the presence of clay sediments and dunes.

Previous research examining burrow location preferences and burrow construction in a range of fossorial mammals has identified connections between sediment properties and both the resulting burrow structure and the likelihood that a burrow is occupied. In particular, at the genus level members of the Geomyidae family harbour preferences for burrows with particular environmental properties; for example, while *G. busarius* prefer sandier loam sediments, *T.bottae* and *P.castanops* prefer clay-loam sediments [31–33]. Additionally, descriptive analysis of other species’ burrows show distinct changes in burrow architecture with changing composition of the sediment in which it is constructed. Individuals of the *Dipodomys* genus (kangaroo rats) construct longer, more complex burrows where there are higher percentages of silt, and *Microtus montanus* (Montane vole) construct deeper, narrower burrows in firmer sediments with more sand [34]. Other studies have described cases where previously suitable habitat has been altered for agriculture (thus changing the sediment properties) and has subsequently been avoided by fossorial mammals [35].

Our results identified that when observed in a single season, burrows in clay sediments are less likely to be occupied. Digging new burrows incurs a cost in any fossorial species, but over the lifespan of the animal this is generally low in comparison to the resultant benefits created by the burrow [36]. The option of constructing a new burrow in clay sediments may be less preferable due to the initially high levels of energy expenditure, despite the stability and high level of protection that one would expect to be conferred by the clay sediments.

In contrast, although neither clay nor dunes on their own had a significant relationship with long term occupancy, the significant interaction term between them indicated that burrows in clay sediments are less likely to remain occupied for longer periods when there are also dunes present, and conversely that burrows in areas with dunes are less likely to remain occupied where clay is also present. The potential benefits one might imagine of sturdier clay soils, and those observed in previous studies [34] may become discounted through disruption in the surrounding landscape created by dunes structures. Similarly, the anticipated benefits conferred by dunes structures (ease of digging due to sand content, and sheltering due to an angled entrance) are both not significantly related to long term occupancy, and where clay sediment is also present any positive effect is mitigated. As dunes do occur frequently across the clay sediment surfaces, the observation that burrows constructed where both are present are less likely to remain occupied has implications for the long term spatial structure of the gerbil populations.

We also observed a significant positive association between a burrow being located in an area of sand and long term occupancy. In *Meriones crassus* burrows constructed in sediments with artificially controlled proportions of sediment types, those constructed in the presence of high proportions of sand result in a higher average temperature of the burrow microclimate compared to other substrate compositions, suggesting sand plays a role in the insulation of the burrow [14]. Our results then may indicate that this insulating property may be key to over-winter or at least multi-season survival for the gerbils, in the face of the drastic temperature fluctuations (hot summers and cold winters) observed in the Pre-Balkhash.

The significant relationship with latitude may reflect the effect of an unmeasured environmental variable that changes along a latitudinal gradient (such as climate), or indeed unmeasured isotropic spatial variation. Gerbil occupancy across the region is known to fluctuate [9], but the spatial correlation drops from around 0.7 at distances of around 20km to around 0.55 at 250km [16]. Further the climate in the pre-Balkhash region correlates closes with latitudinal gradient [37]. Therefore both latitudinally varying factors and latitudinally-independent asynchronies may both play a role here.

The positive relationship between the presence of trees and static occupancy is perhaps suggestive of a necessary level of moisture (supporting both tree growth and occupancy) and/or protection from excess heat through shading, the latter fitting with previous observational records [17]. In addition, an experimental study of the vole, *Microtus pinetorum*, identified preference for moderate soil moisture, likely to be a balance between digging ease and risk of flooding [38]. Albeit in different ecosystems, small mammals have been shown to positively impact the dispersal of tree seeds throughout the environment [39]. Bioturbation of the soil by gerbils as they burrow may also aid plant growth by enabling redistribution of micronutrients in the soil [40]. Thus, the gerbils themselves may increase the presence of trees, possibly creating a positive feedback system or so called “fertile islands” [15].

Lower occupancies in spring in particular are likely simply to reflect the natural fluctuations of gerbil populations, where numbers are generally highest in autumn (e.g., [9]), following which reproduction ceases and numbers decline over the winter. Furthermore the lower occupancies in the later trapping seasons may also simply reflect the general decline in gerbil numbers across the entire region during this period. Field observations suggest a gerbil population crash occurred around 2011, but that gerbil numbers remained higher in the Northern region (also likely to influence the relationship with latitude).

### Fleas

Although several flea species in this system are potential plague vectors, *Xenopsylla spp.* are the most important [41], (with *Coptopsylla lamellifer* playing a role in winter transmission) and are the dominant species in our data set. The different species themselves differ in their seasonal dynamics and details of their habitat requirements. Here, latitude, season and tick burden associated with a burrow were identified to be significantly related to the burden of fleas in the burrow.

The effect of season is likely to be linked with the associated abiotic environmental changes. There may also be an impact in the changing vegetation through the seasons, with the vegetation cover determining shade, temperature and soil moisture at scales affecting the fleas [42]. Here, rodents trapped in Autumn 2012 had a lower flea burden. This likely reflects the dip in flea numbers over the colder months typically observed in the *Xenopsylla* life cycle, combined with a general decline in host abundance. In general these relationships reflect the well documented effects of season on *Xenopsylla*. Similarly to many insect species, external temperatures have direct effects on the metabolic and therefore developmental processes of fleas, and their survival is reduced at low humidities [42, 43]. For fleas in particular, as developmental stages occur off host, their developmental rate and population dynamics are controlled greatly by abiotic factors [44].

That tick burden is a significant predictor of flea burden suggests that there may be burrow conditions not monitored here that create conditions permissive of a wide variety of ectoparasites and that are conducive to the spread of plague. Alternatively, since the flea and tick measurements relate to numbers found on individual gerbils, the association may reflect characteristics of these individuals rather than the burrows in which they were trapped. Certainly, flea infestation is known to affect the metabolism of the mammalian host [45]. Further, alterations to the host behaviour and immune response by an initial ectoparasite infestation may then make the host more susceptible to multiple ectoparasite infestation [46], even before considering the immunological effects of infection by *Y.pestis* to either the gerbils or fleas. Discerning cause and effect here is difficult, but there is certainly an indication that the wider ectoparasite and invertebrate communities should be considered in order to properly characterise the dynamics of this plague system. Indeed, several authors report transmission or isolation of *Y.pestis* in *Ixodidae* (*Rhipicephalus sp.* and *Hyalomma sp.*) and *Argasidaee* ticks (in particular, *Ornithodoros tartakovsky*) in Central Asian desert foci [47–49], and this was also the case in the present study [50].

Finally, migrating fleas may provide insight into the specific role of fleas in determining the timing and size of epizootics. A theoretical study based on the American prarie dog plague system identified a potential role for migrating fleas in enzootic persistence between epizootic events [51]. However, data collected here on fleas aspirated directly from burrows was not considered to be sufficiently consistent for use in a statistical analysis equivalent to that for fleas collected directly from gerbils.

### A perfect burrow for gerbils, fleas and *Yersinia pestis*

At the most basic level, for *Y.pestis* epizootic events to spread and be sustained, both the mammalian and insect host must be present across the landscape. This would then suggest that a burrow in the northern region, with surrounding vegetation, and perhaps located in a sandy areas, would be at the highest risk for plague presence given the significant positive relationships between these properties and host and vector presence identified here. Plague prevalence during the study period was not high enough to test this directly. There may also be other factors that need to be present or absent for a burrow to be permissive of *Y.pestis* itself. The question also remains of where the bacterium survives in interepizootic periods (or indeed, through gerbil population crashes), which may itself be controlled by abiotic environmental conditions. Overall, this study gives finer resolution to our ability to predict distributions of both of the key host and vector species in this system, so improving our ability to predict plague epizootic occurrence and behaviour. As a starting point to investigate this, a closer analysis could be conducted of environmental factors linked to specific burrow transition events from season to season.

## Supporting Information

S1 File Appendices. Further detail on laboratory and statistical procedures. Table I, Landscape descriptor codes used to record landscape description in the field. Figure A, Normalised log proportion of occupied burrows for each of the landscape descriptions, in rank order. Figure B, Frequency of single letter landscape descriptor codes across 3 groups of proportion of occupied burrows. Figure C, Median flea index associated with burrows possessing each of the unique landscape descriptors where a flea index had been recorded. Figure D, Frequency of each of the landscape code across three strata of flea index scores. Table II, Landscape descriptor codes included in predictive factors. Figure E, Predicted proportion of seasons occupied for burrows with and without dunes or clay, and combinations thereof. Figure F, Plots showing coefficient estimates and P values for the model using the sector and burrow random effects and for the iterative model using sector random effects but random samples of burrows. Figure G, Plots showing coefficient estimates and P values for the model using the sector and burrow random effects and for the iterative model using sector random effects but random samples of burrows. Figure H, Flea index and tick index scores, with predictions from the minimal model.
(DOCX)
