# Supplementary material for: The Perfect Burrow, but for What? Identifying Local Habitat Conditions Promoting the Presence of the Host and Vector Species in the Kazakh Plague System
Source: PLoS One. 2015 Sep 1;10(9):e0136962. doi: 10.1371/journal.pone.0136962 (PMC4556633; doi:10.1371/journal.pone.0136962)
Supplement: S1 File — Further detail on laboratory and statistical procedures. Table I, Landscape descriptor codes used to record landscape description in the field. Figure A, Normalised log proportion of occupied burrows for each of the landscape descriptions, in rank order. Figure B, Frequency of single letter landscape descriptor codes across 3 groups of proportion of occupied burrows. Figure C, Median flea index associated with burrows possessing each of the unique landscape descriptors where a flea index had been recorded. Figure D, Frequency of each of the landscape code across three strata of flea index scores. Table II, Landscape descriptor codes included in predictive factors. Figure E, Predicted proportion of seasons occupied for burrows with and without dunes or clay, and combinations thereof. Figure F, Plots showing coefficient estimates and P values for the model using the sector and burrow random effects and for the iterative model using sector random effects but random samples of burrows. Figure G, Plots showing coefficient estimates and P values for the model using the sector and burrow random effects and for the iterative model using sector random effects but random samples of burrows. Figure H, Flea index and tick index scores, with predictions from the minimal model. (DOCX) [file pone.0136962.s001.docx]

**Appendix**

*Generating landscape factors for occupancy model*

In the field, single letter/number codes were used to record the presence of up to 15 landscape properties surrounding the burrow (see reference to Pogrebinsky (1963) in the main text). The codes recorded, and the landscape properties they represent, are given in Table 1 below. The burrow description would then be made up of a combination of these, using as many as were necessary to fully describe the site, e.g. a burrow in a dry river bed bottom with non-stabilised dunes would be recorded as A7.

*Table I. Landscape descriptor codes used to record landscape description in the field.*

| **Topographical features** | | **Sediment properties** | |
| --- | --- | --- | --- |
| **Landscape code** | **Landscape aspect represented** | **Landscape code** | **Landscape aspect represented** |
| A | Dry river bed bottom | 1 | Sand |
| B | Slope of dry river bed | 2 | Sandy Loam |
| C | Alluvial Plain | 3 | Clay Loam |
| D | Low slow of dune on alluvial plain | 4 | Smectite Clay |
| E | High slope of dune on alluvial plain | 5 | Solonchak (salt accumulation in soil due to evaporation of water) |
| F | Top of dune in alluvial plain | 6 | Takir (water logged) |
| K | Depression in alluvial plain | 7 | Non Stabilised dunes |
|  |  | 8 | Stabilised Dunes |

The resulting 171 unique landscape descriptions generated using this process represented a great depth of detail about the environment, but one that would be difficult to utilise statistically without losing power. Whilst there are natural groupings within the descriptors (i.e. D, E and F all describe properties of dunes), grouping them simply by personal judgment, without an initial examination of their relationships to the outcome variables, may have ignored potential intricacies of the relationships between environment and outcome. For example, it could be that a burrow on the top of a dune has a lower chance of being occupied than that on the side of a dune, arguing against their combination in a ‘dune’ factor. As such an initial exploratory analysis was performed to generate summary descriptions of the relationships between each of the above descriptors and the outcome variables (occupancy and flea burden) to identify those that could be grouped and would be useful to use in the final analysis. Here occupancy is taken to be the outcome variable in the first example.

The proportion of all occupied burrows represented by each of the 171 unique landscape descriptions generated using the codes above was calculated, and then normalised by dividing by the number of burrows recorded of this description. Plotting this data made it quite clear that there were groups of landscape descriptions that represented similar proportions of all occupied burrows – implying that they had some shared characteristic that made them similarly likely to be associated with an occupied burrow (Figure a).


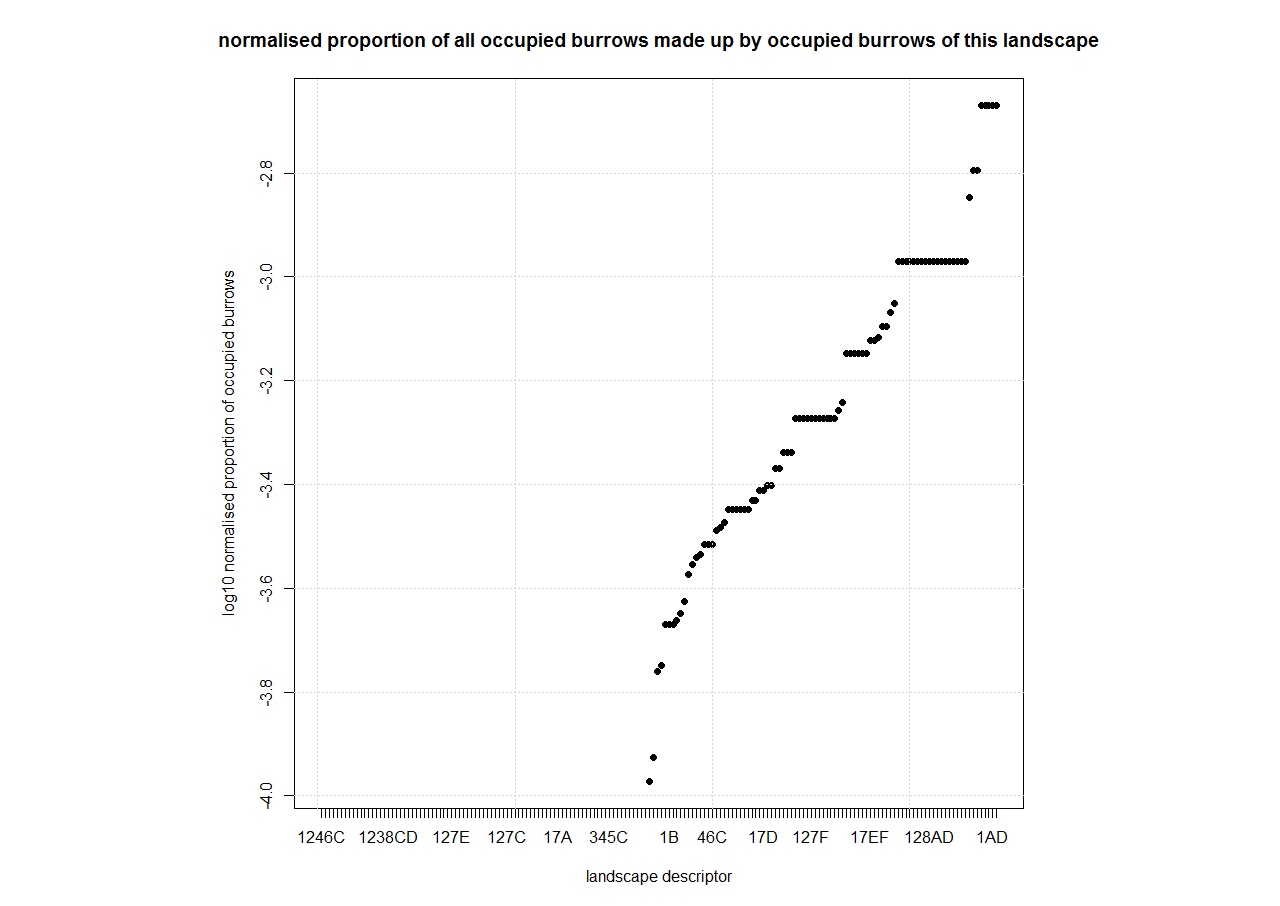


*Figure a. Normalised log proportion of occupied burrows for each of the landscape descriptions, in rank order.*

To identify possible links between landscape descriptors and occupancy, the landscape descriptions (i.e. the horizontal axis labels in Figure a) were divided into approximate thirds reflecting the occupancy levels they were associated with, i.e. the landscape descriptions associated with the lowest, medium and highest levels of occupancy were grouped together. The level of representation of each of the above descriptor codes was calculated for each of the groups, in order to identify descriptors that had changing proportions across the different occupancy levels, asking the question: are any of the descriptor codes notably more or less common in high or low occupancy levels?

This was plotted as a “heat map”, with colour changes from white through to black (low to high) (Figure b).


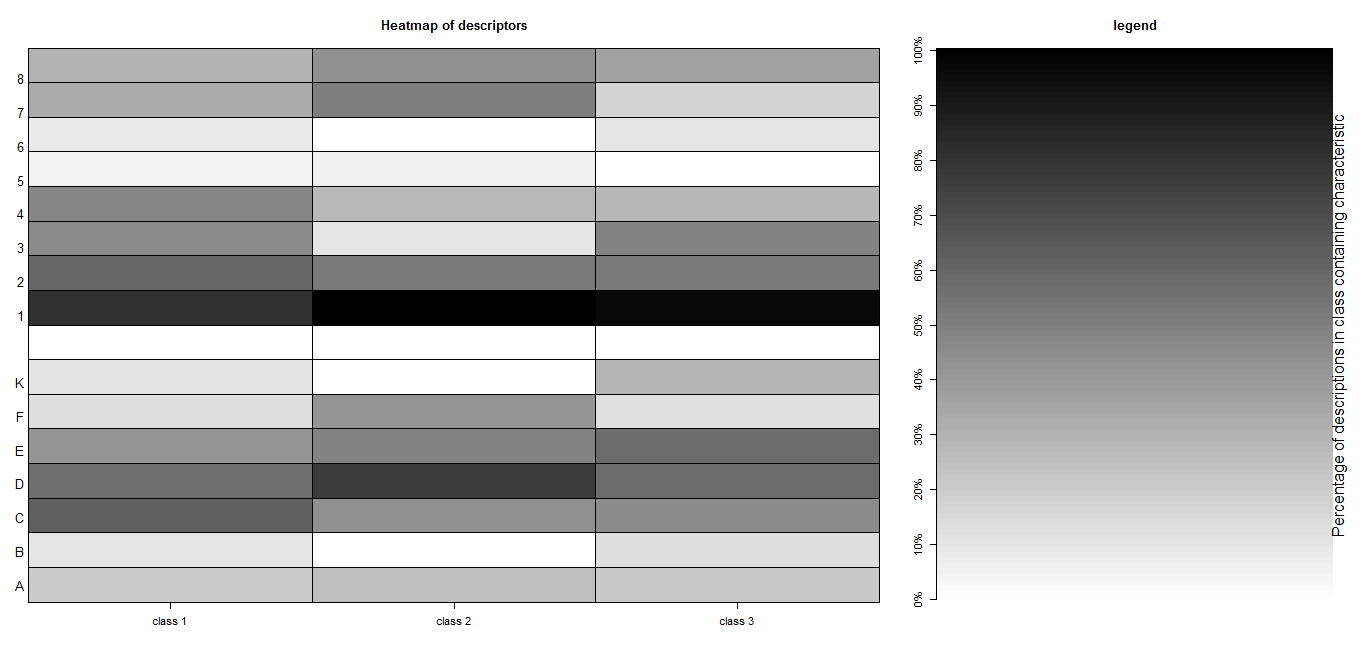


*Figure b. Frequency of single letter landscape descriptor codes across 3 groups of proportion of occupied burrows (class 1 representing the lowest proportion of occupied burrows, and class 3 the highest proportion of occupied burrows).*

So for example here: the descriptors D, E, F, 7 & 8 all describe properties relating to dunes: either the position of the burrow relative to the slope of the dune, or the stability of the dune. They may potentially be useful to consider together as a predictive factor, if there is a relationship between dunes in general and the probability of occupancy, rather than specific dune properties. However, if the relationships with occupancy differ between these descriptors, they may need to be included as separate factors.

If we examine the transitions from high to medium and medium to low in figure b, then all five of the above descriptors showed can increase from the low to the medium group (each representing roughly a 5% difference in the percentage representation) and three of the five a decrease from the medium to the high group (again representing approximately a 5% difference in representation. On the basis of this consistency, we proceed with the environmental term “dunes”, with presence recorded for any burrow with a description containing any of the descriptors D,E,F,7 or 8.


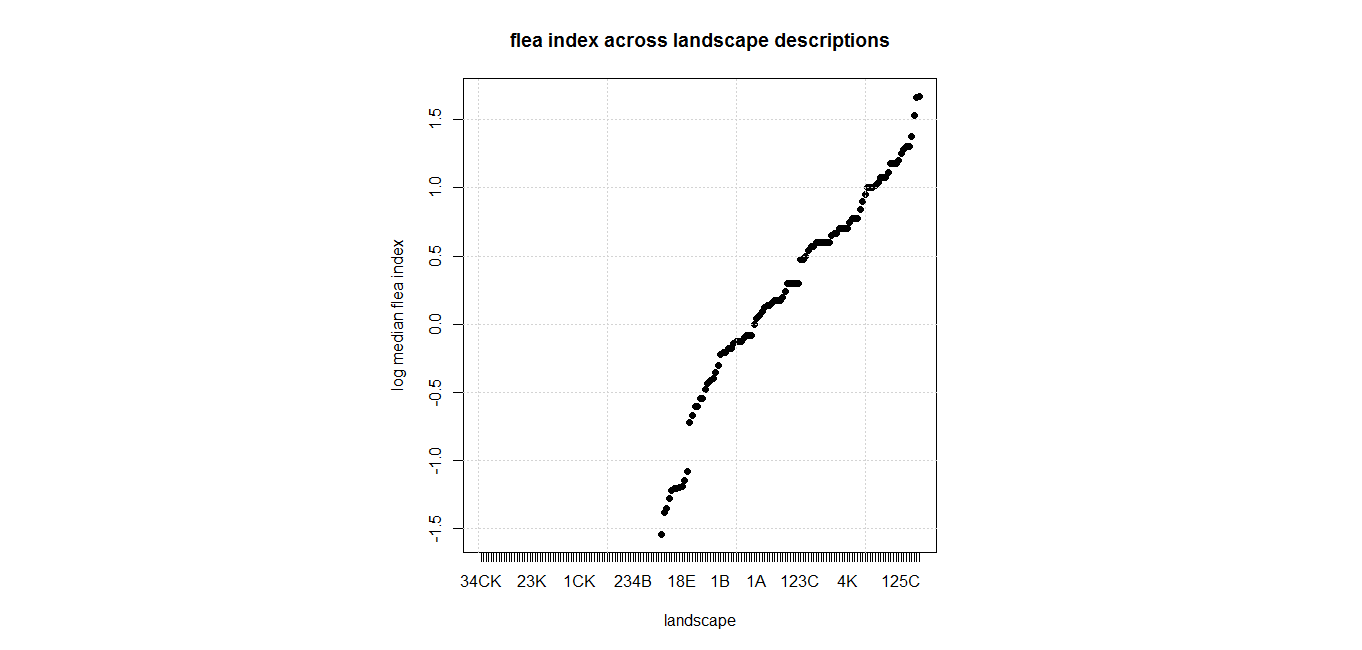
*Generating landscape factors for flea model* A similar method was applied to the flea data. As the flea index records are a continuous variable, each landscape type was simply plotted against the median flea index associated with records possessing this landscape type, as in Figure c.

*Figure c. Median flea index associated with burrows possessing each of the unique landscape descriptors where a flea index score had been recorded.*

In a similar way to occupancy, a heat map was produced showing the frequency of each of the landscape codes across increasing levels of flea index (Figure d).


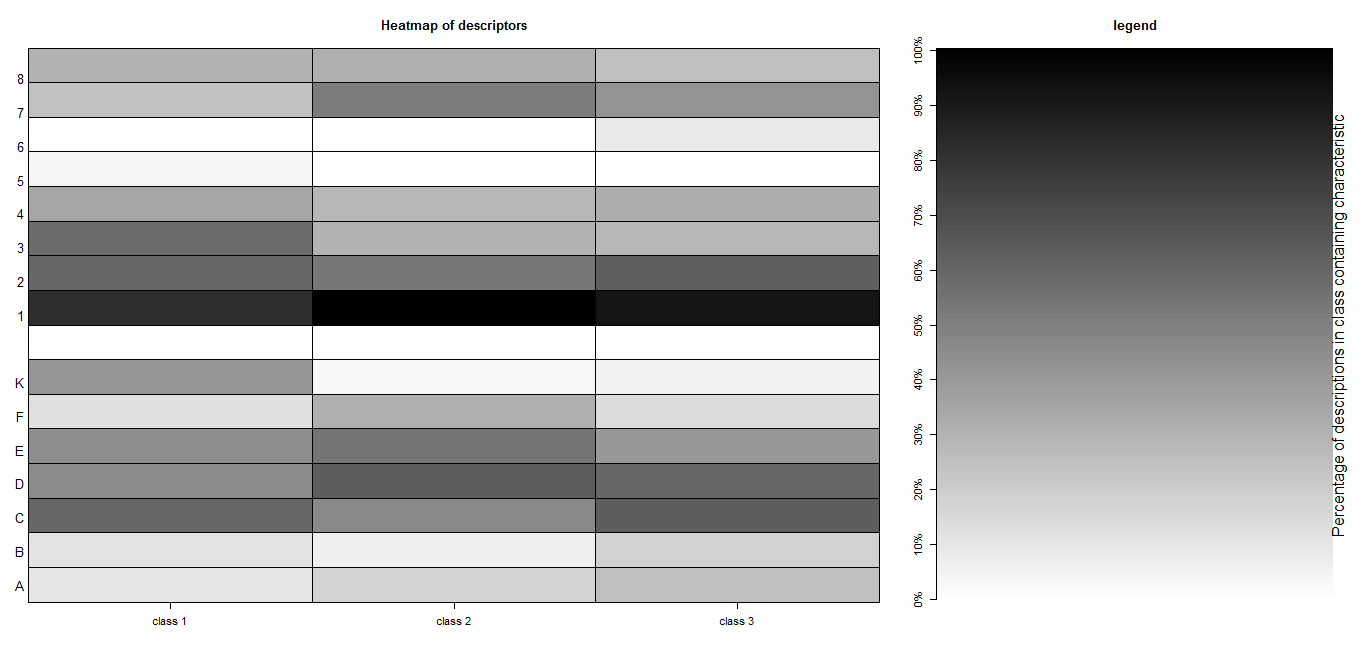


*Figure d. Frequency of each of the landscape codes across three strata of flea index scores. The rows labelled 7&8 and C-F represent descriptors relating to dunes, and are an example of potential descriptors for inclusion in predictive factors.*

The results generated in both cases were then used to construct the landscape descriptors as indicated in Table 2 (main text). The factors used in each of the models are then described in Table 3 (main text).

**Landscape factors**

The following landscape factors were used in the analysis (their inclusion in models is detailed in the main text in Table 2. The table below details the specific descriptors that would be present to classify a burrow as having the associated landscape factor.

*Table II. Landscape descriptor codes included in predictive factors.*

| **Factor** | **Descriptor code determining inclusion** |
| --- | --- |
| Sand | 1 |
| Clay | 4 |
| Loam | 2,3 |
| Solonchak/Takir | 5,6 |
| Dunes | D,E,F,7,8 |

**Analysis of the interaction term**

The long term occupancy minimal model ( long term occupancy ~ latitudinal position + sand + dunes * clay ) contains an interaction term between the presence of alluvial sediments and dunes. The interaction term was investigated further by generating predictions of the outcome variable (proportion of seasons a burrow is occupied) given presence/absence/combinations thereof of the 2 predictors. This is shown in Figure e.

*
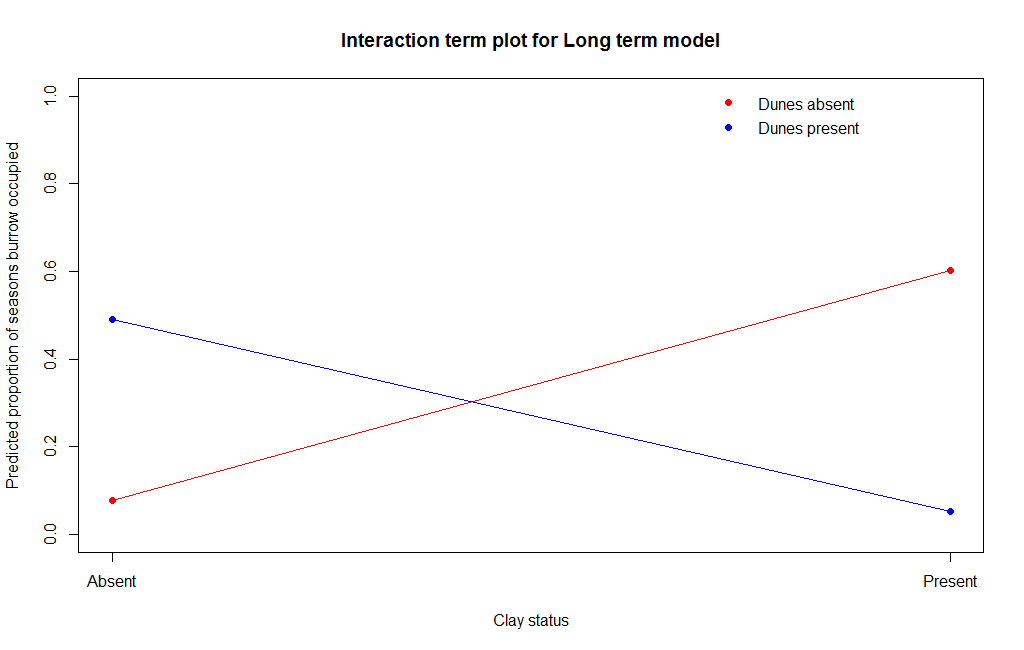
*

*Figure e. Predicted proportion of seasons occupied for burrows with and without dunes or clay, and combinations thereof.*

**Results from iterative repeats of models to test robustness of random effects structure**

As an additional confirmation of the results produced by the model with the nested random effects structure, they were compared with those from models generated by selecting at random just one observation from each burrow and repeating this 100 times (Telfer et al., 2010). As Figure f shows, there were no significant deviations (i.e. coefficients becoming positive rather than negative, or losing of significance) from those generated by the mixed effects model, supporting the original model.

*Occupancy*

*
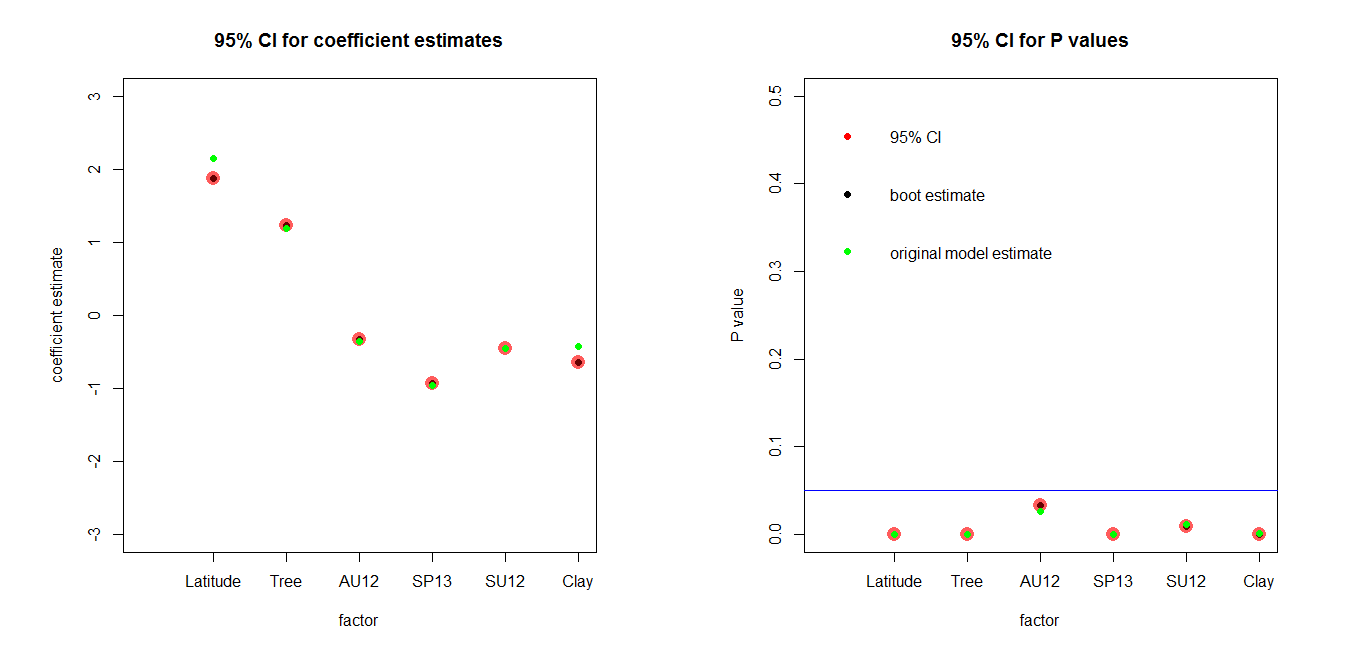
*

*Figure f. Plots showing coefficient estimates and P values for the model using the sector and burrow random effects (green points) and for the iterative model using sector random effect but random samples of burrows. These iterative results are plotted in black (showing the average result) and red (showing the range of the 95% confidence intervals). There is a high degree of overlap between the values from the iterative sampled models and the random effects model, suggesting agreement between the random effects model and the results from the iterative sampling.*

*Xenopsylla* burden

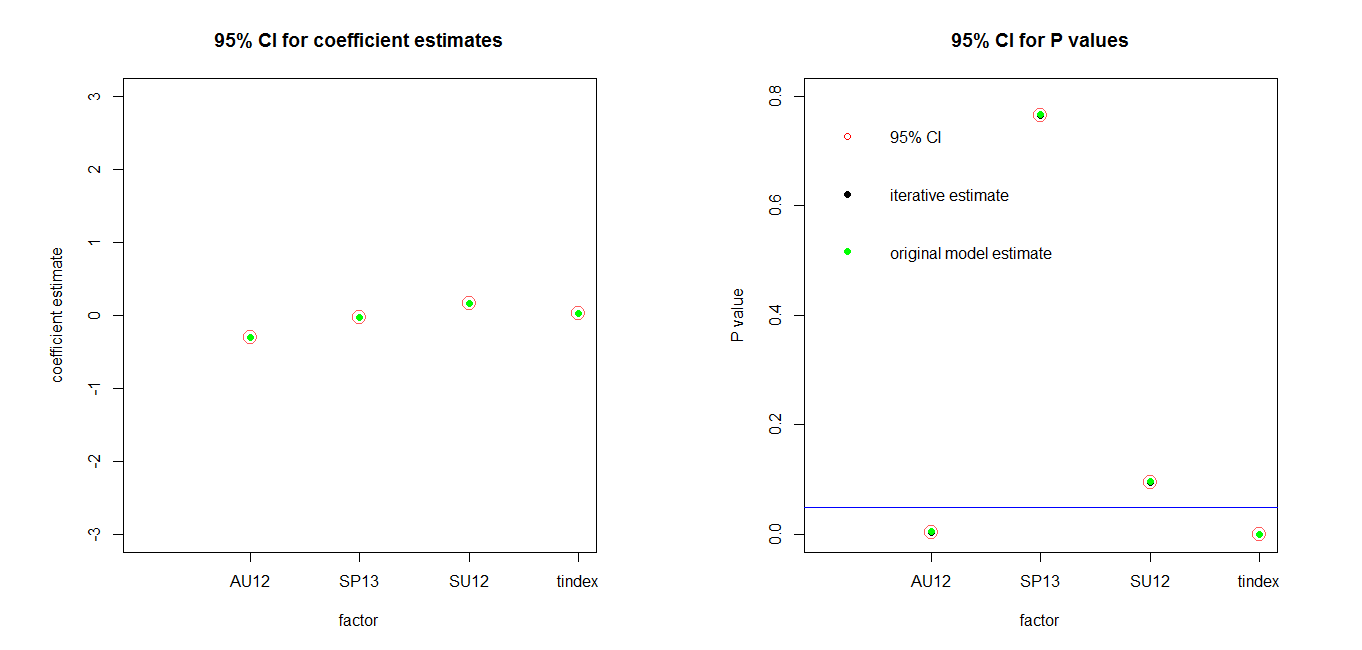


*Figure g. Plots showing coefficient estimates and P values for the model using the sector and burrow random effects (green points) and for the iterative model using sector random effect but random samples of burrows. These iterative results are plotted in black (showing the average result) and red (showing the range of the 95% confidence intervals). Again the high degree of overlap between the results suggests agreement between the random effects models and models from the iterative sampling process.*

**Flea and tick burden relationship - plot of data and predicted values**


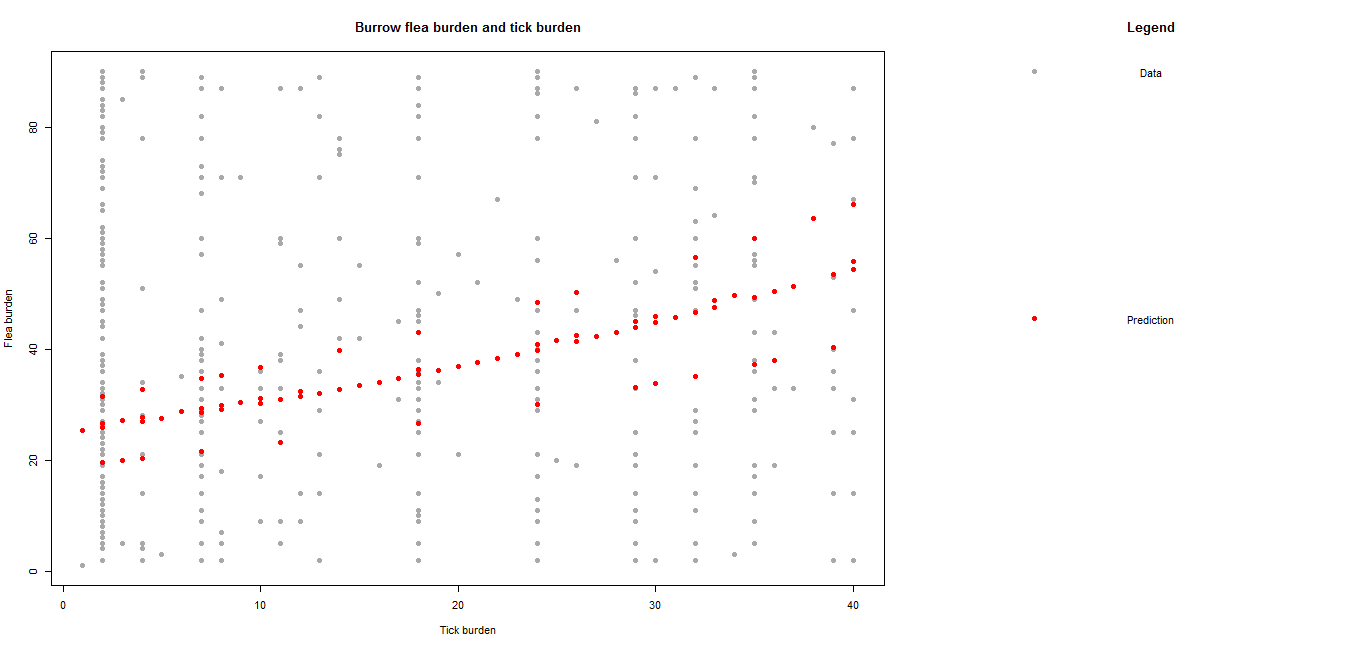


*Legend*: ● Collected Data

● Predictions from GLMM

*Figure h. flea index and tick index scores, with predictions from the minimal model shown in red.*

*Laboratory procedures*

Mammals trapped in the field were brought to the Bakanas anti-plague station where their mensurations, sex, sex condition, ectoparasite burden and diversity, and plague status were recorded. Processing consisted of combing the fur for ectoparasites, sampling organs (kidney, spleen, liver, heart, lungs) which were stored in ethanol for further screening, and *Y.pestis* isolation attempt from spleen, liver and blood samples. Culture attempts were conducted using Hottinger agar incubated at 28C for 48 hours. Haemaglutination tests (for PHA and IPHA) were performed to test for seropositivity of the gerbils by measuring F1 antibody production. Stomach contents were excised and trace element concentration recorded. All rodent carcasses were then disinfected in a bath of dethol for 12 hours and incinerated.
